# The Accuracy of Breast Cancer Diagnostic Tools

**DOI:** 10.7759/cureus.51776

**Published:** 2024-01-07

**Authors:** Batool S Alotaibi, Rahaf Alghamdi, Sadeem Aljaman, Reem A Hariri, Lama S Althunayyan, Batool F AlSenan, Areej M Alnemer

**Affiliations:** 1 Medicine and Surgery, Imam Abdulrahman Bin Faisal University, Dammam, SAU; 2 Pathology, Imam Abdulrahman Bin Faisal University, Dammam, SAU

**Keywords:** ultrasound (us), magnetic resonance imaging (mri), mammography, breast cancer pathology, radiological modalities, breast disease

## Abstract

Background

Breast cancer (BC) remains a significant health concern, leading to illness and death among women globally. It is essential to detect BC early using imaging techniques that accurately reflect the final pathology, guiding suitable intervention strategies.

Objectives

This study aimed to evaluate the agreement between radiological findings and histopathological results in BC cases.

Methods

We conducted a retrospective review of breast core needle biopsies (CNBs) in women over a six-year period (2017-2022) at Imam Abdulrahman Bin Faisal University, Dammam, Saudi Arabia. The pathological diagnoses were compared with the findings from preceding radiological investigations. We also compared the tumour sizes in the resection specimens with their radiological counterparts.

Results

A total of 641 cases were included in the study. Ultrasound (US), mammography, and magnetic resonance imaging (MRI) yielded diagnostic accuracies of 85%, 77.9%, and 86.9%, respectively. MRI had the highest sensitivity at 72.2%, while US had the lowest at 61%. MRI provided the best agreement with the final resected tumor size. By contrast, mammography tended to overestimate the size (41.9%), and US most frequently underestimated it (67.7%). The connection between basal-like molecular subtypes and the Breast Imaging Reporting and Data System (BIRADS)-5 classifications was only statistically significant for MRI (p = 0.04). The luminal subtype was more likely to show speculation in mammography. Meanwhile, BIRADS-4 revealed a considerable number of benign pathologies across all the three modalities.

Conclusions

MRI demonstrated the highest accuracy, sensitivity, specificity, and positive predictive value (PPV) for diagnosing and estimating the tumor size. Mammography outperformed US in terms of sensitivity and yielded the highest negative predictive value (NPV). US, meanwhile, offered superior specificity, PPV, and accuracy. Therefore, combining these diagnostic methods could yield significant benefits.

## Introduction

Breast cancer (BC) is the most prevalent cancer among women, constituting 24.2% of all cancer cases [1]. An estimated 2.3 million new cases were diagnosed globally in 2020 [2]. Various imaging methods, including ultrasonography (US), mammography, and magnetic resonance imaging (MRI), are often used to diagnose BC [3]. These images not only aid in identifying the lesion but also provide significant data, such as tumor size, critical for staging and treatment and for tracking the response to neoadjuvant therapy [4].

Each imaging technology has distinct characteristics and restrictions. For instance, Abedi et al. found that MRI is particularly effective in estimating tumor size during neoadjuvant therapy [5]. If radiological features suggest possible BC, a core needle biopsy (CNB) is necessary for a definitive diagnosis since it is considered the gold standard [6].

The interpretation of radiological images is classified based on the level of suspicion using the Breast Imaging Reporting and Data System (BIRADS) [7]. This classification helps decide whether further histopathological examination (HPE) is required. Research supports MRI’s superior sensitivity for the early detection of suspicious breast masses, outperforming mammography and US [7,8].

BCs are classified into molecular subtypes, such as luminal, HER-2 enriched, and basal, based on the expression of different receptors, as determined by immunohistochemistry (IHC) testing [9]. This test offers significant prognostic value. The ability to correlate various radiological features with receptor expression enhances prognosis prediction.

Notably, a discrepancy between the radiological and pathological tumor size can lead to inaccurate staging. Hence, it is important to identify the imaging method that most accurately approximates the tumor size. Maintaining high concordance between radiological and pathological parameters is crucial for making precise management decisions [10].

This study intends to establish a correlation between radiological and histopathological findings in relation to BIRADS scoring, final diagnoses, and size measurement of breast masses. Furthermore, it attempts to examine the relationship between various radiological findings and the molecular subtypes of BC.

## Materials and methods

Institutional research ethics approval was received from Imam Abdulrahman Bin Faisal University Institutional Review Board (IRB) (IRB-UGS-2022-01-436). This study targeted cases of breast lumps examined using CNB from the beginning of January 2017 to the end of December 2022 at Imam Abdulrahman Bin Faisal University, Dammam, Saudi Arabia. It only considered female patients who had at least one breast imaging test, such as US, mammography, or MRI, before the CNB. Any cases diagnosed with inflammatory breast conditions, such as mastitis, cysts, granulomas, or abscess walls, via histopathology or imaging were excluded from the study. Moreover, cases classified as BIRADS 0 and 6 were not included when determining the accuracy rate. BIRADS scores range from 1 to 5 and are used to categorize breast imaging results. Scores 1, 2, and 3 indicate benign findings, 4 signifies suspicious findings, and 5 represents malignant results.

Preoperative imaging records performed immediately prior to the core biopsy were reviewed. Patients who underwent a core biopsy were categorized into three distinct groups: Group 1 underwent US, Group 2 had mammography, and Group 3 opted for MRI. Specific imaging characteristics of the breast mass subsequently sampled by pathology were extracted from the hospital’s imaging report system. These features encompass mass size, margin, calcifications, and internal vascularity. The results were classified according to the BIRADS score into three categories: benign (BIRADS 1, 2, 3), suspicious (BIRADS 4), and malignant (BIRADS 5).

Pathological findings were also analyzed for cases with available CNB and subsequent surgical excision reports. These reports were reviewed to collect data on diagnoses, tumor sizes, histopathological types, and molecular subtypes. BC instances were categorized into a variety of subtypes: luminal (ER and/or PR positive, irrespective of HER-2 status), basal (negative for ER, PR, and HER-2) and HER-2 enriched (only positive for HER-2 receptor).

Statistical analysis

We used the IBM SPSS Statistics for Windows, version 27 (released 2019; IBM Corp., Armonk, New York, United States) to analyze the data statistically. A chi-squared test (χ2) was performed on qualitative data, while the Mann-Whitney test was used for non-parametric quantitative variables. A p-value less than 0.05 defined statistical significance.

## Results

Patients’ age distribution for each imaging modality

**Figure 1:**
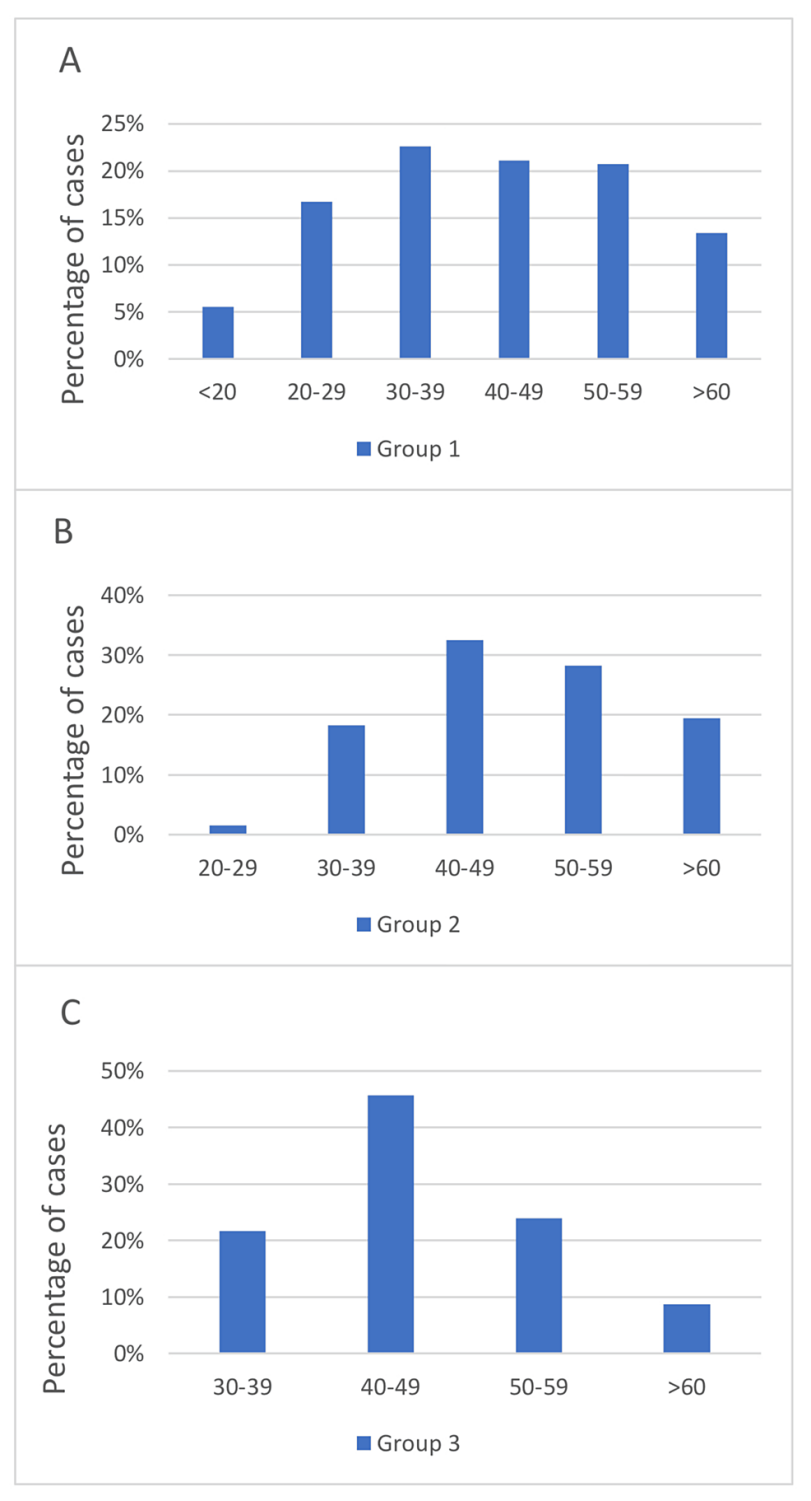
Bar charts showing the age distribution for ultrasound (1A), mammography (1B), and MRI (1C).

The study included 641 CNBs, divided into Group 1, which includes those who underwent CNB and US with 508 cases (aged 13-88, average age 42.26 ± 14.72); Group 2, which includes those who underwent CNB and mammography with 323 cases (aged 22-77, average age 49.43 ± 10.78); and Group 3, which includes those who underwent CNB and MRI with 46 cases (aged 30-65, average age 46.7± 8.57), as can be seen in Figure 1A-1C.

Accuracy testing

Group 1 (US)

Of the 508 cases analyzed, 107 were radiologically defined as malignant, 355 as suspicious, and 46 as benign per BIRADS scores. From these, pathology diagnoses identified 154 cases as malignant (see Table 1). Thus, the diagnostic accuracy of US was characterized as follows: 61% for sensitivity, 96.3% for specificity, and 85% each for positive predictive value (PPV), negative predictive value (NPV), and overall diagnostic accuracy.

**Table 1:** Correlation between the histopathology results and BIRADS diagnosis of the three radiologic modalities (ultrasound, mammography, and magnetic resonance imaging).

Radiologic modalities		Histopathological diagnosis		χ2	p-value
		Benign	Malignant		
Ultrasound	Benign	42 (11.9%)	4 (2.6%)	13.37	< 0.001
	Suspicious	299 (84.5%)	56 (36.4%)		
	Malignant	13 ( 3.7%)	94 (61%)		
Mammography	Benign	30 (16.6%)	5 (3.5%)	32.09	< 0.001
	Suspicious	140 (77.3%)	43 (30.3%)		
	Malignant	11 (6.1%)	94 ( 66.2%)		
Magnetic resonance imaging	Benign	1 (3.6%)	1 (5.6%)	25.44	<0.001
	Suspicious	26 ( 92.9%)	4 ( 22.2%)		
	Malignant	1 ( 3.6 %)	13 ( 72.2 %)		

Group 2 (Mammography)

The study identified 105 malignant radiological cases, 183 suspicious cases, and 35 benign lesions. Pathological analysis unveiled 142 malignant lesions (see Table 1). The measures of sensitivity, specificity, PPV, NPV, and diagnostic accuracy were 66.1%, 93.9%, 89.5%, 77.9%, and 81.7% respectively.

Group 3 (MRI)

MRI classified only 14 cases as malignant, 30 as suspicious, and two as benign. Meanwhile, CNBs revealed 18 malignant cases, as demonstrated in Table 1. Therefore, MRI’s sensitivity, specificity, PPV, NPV, and accuracy rate were 72.2%, 96.4%, 92.8%, 84.3%, and 86.9%, respectively.

Significance of BIRADS-4

**Figure 2:**
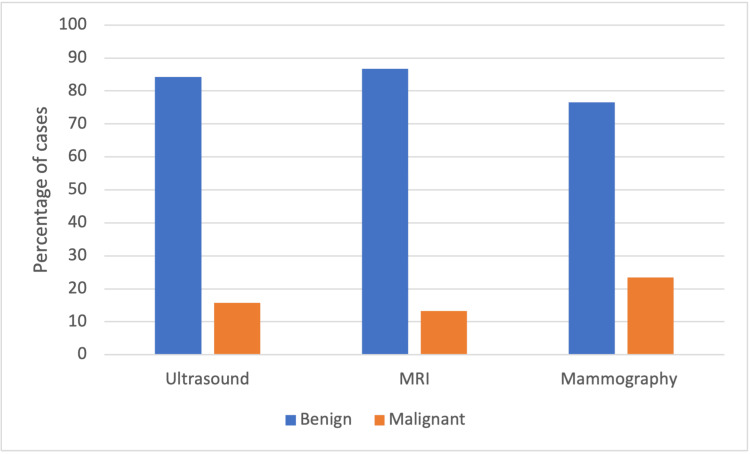
Correlation between the histopathology results and BIRADS-4 diagnosis of the three radiologic modalities.

A total of 355, 183, and 30 cases were identified as suspicious (BIRAD-4) through US, mammography, and MRI, respectively as shown in Table 1 and Figure 2. Pathology, however, revealed benign diagnoses in 299 (84.2%), 140 (76.5%), and 26 (86.7%) of these respective cases.

Estimation of tumor size

**Figure 3:**
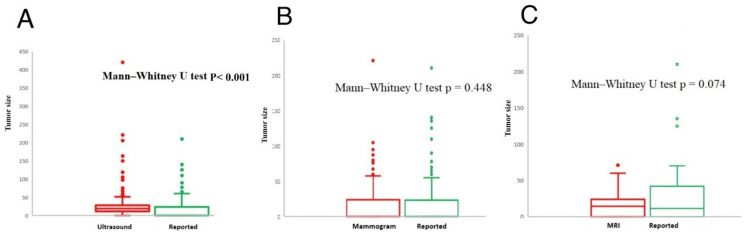
The mean, median, and range between the size measurements of ultrasound (3A), mammography (3B), and MRI (3C) and the size of mass excision.

Tumor size estimation across the three imaging modalities was correlated with measurements taken at excision, using mean, median, and range comparisons (see Figure 3A-3C). A significant discrepancy was observed for US readings (n = 220), with mean US measurement = 25.23 ± 8.44 mm, compared to 32.85 ± 27.85mm at excision (p < 0.001). Most cases revealed an underestimation of size (n = 149, 67.7%), with an overestimation in 61 cases (27.7%) and accurate estimates in just 10 cases (4.5%), as demonstrated in Figure 3A. In the 62 cases that underwent mammography followed by excision, no significant difference was found in the tumor size measurement (p = 0.448).

The average size on mammography was 31 ± 25.65 mm, while it was 36 ± 33.8 mm after excision. An underestimation was detected in 34 cases (54.8%), overestimation occurred in 26 cases (41.9%), and there was concordance in two cases (3.2%), as shown in Figure 3B. Regarding MRI, no significant size difference was observed (n = 19, p = 0.074). The mean size on MRI was 23 ± 19 mm, while it measured 44.98 ± 46.2 mm upon excision. Among the cases, underestimation occurred in 10 instances (52.6%), overestimation in seven cases (36.8%), and there was concordance in two cases (10.5%), as shown in Figure 3C.

Correlation between IHC-based molecular subtypes of BC and radiologic findings

IHC-Based Molecular Subtypes of BC and BIRADS Scoring

In US, there were 148 BC cases, subdivided into 118 luminal, 15 HER-2 enriched, and 15 basal types. Mammography identified 134 BC cases, which included 106 luminal, 15 HER-2 enriched, and 13 basal-like types. Of the 17 cases detected by MRI, 14 were luminal, two were HER-2 enriched, and one was a basal-type case. For both US and mammography, the relationship between molecular subtypes and BIRADS classifications was non-significant statistically (with p-values of 0.176 and 0.282, respectively).

In the US group, the luminal type of BC was categorized as benign in four cases (3.4%), suspicious in 43 cases (36.4%), and malignant in 71 cases (60.2%). The majority of cases with the HER-2 enriched type were interpreted as malignant (12 cases, 80%), with suspicious results observed in three cases (20%). The basal type was considered suspicious nine times (60%) and malignant six times (40%).

In terms of mammography, the luminal type was categorized as benign in five cases (4.7%), malignant in 72 cases (67.9%), and suspicious in 29 cases (27.4%). The HER-2 enriched type was found to be malignant in 11 cases (73.3%) and suspicious in four cases (26.7%). The basal type was determined as malignant in six instances (46.2%) and suspicious in seven cases (53.8%).

In the MRI cases, a significant statistical correlation was noticed between BIRADS scoring and IHC-based molecular BC subtypes (p = 0.04). However, the limited number of cases precludes a definitive conclusion. The luminal type was generally suspicious (21.4%) or malignant (78.6%). The HER-2 enriched type was evenly split between benign and suspicious cases, with one case each. The basal-like subtype was entirely malignant (100%, n = 1).

IHC-Based Molecular Subtypes and Tumor Calcification, Internal Vascularity, and Margin Spiculation

In US, calcifications, internal vascularity, and spiculated margins were observed 11, 48, and 100 times, respectively. There was no significant statistical relationship between IHC-based molecular types and these three suspicious radiological features. Most calcifications were linked with the luminal subtype (72.7% or eight instances), compared with 18.2% and 9.1% for the HER-2 and basal subtypes, respectively (p = 0.655). The luminal subtype accounted for the highest proportion of internal vascularity sightings (77.1% or 37 times), while this was observed only 16.7% and 6.2% of the time in HER-2 and basal subtypes, respectively (p = 0.131). Spiculated margins were primarily associated with the luminal subtype (78 occurrences, 78%), compared with HER-2 enriched (10 instances, 10%) and basal subtypes (12 cases, 12%), resulting in a p-value of 0.74.

The mammography group showed a significant link between luminal BC and spiculated margin, evidenced in 65.9% (27 occurrences) of cases compared to 12.2% in HER-2 enriched and 21.9% in basal subtypes (p = 0.023). Internal vascularity, another radiological feature, was not significantly observed, with only two instances in luminal and one in basal (p = 0.361). In addition, calcifications were noted 49 times in luminal, 10 times in HER-2 enriched, and eight times in basal-like occurrences (p = 0.323). Unfortunately, due to a limited number of cases, these three features were not analyzed for MRI.

## Discussion

The early diagnosis of BC relies heavily on the accuracy of imaging modalities. To gain a more thorough understanding, we conducted an extensive and comprehensive analysis of the diagnostic capabilities of US, mammography, MRI, and tissue biopsy in relation to BC.

A total of 508 cases underwent US and biopsy (Figure 1A). The study found a correlation between the BIRADS score and HPE, showing that 91.3% of BIRADS-2 and 3 cases were benign (Table 1). This aligns with Bello et al.’s study, which found benign diagnoses in 100% and 87.3% of BIRADS-2 and 3 cases, respectively [11]. Of the BIRADS-4 cases in our study, 15.8% were malignant, which lies between the percentages reported in Bello et al.’s study (81.1% malignancy) [11] and Lehman et al.’s study (8.6% malignancy) [12]. In terms of BIRADS-5, our study found that 87.9% were malignant, comparable to 83.3% and 87.3% reported by Bello et al. and Lehman et al., respectively [11,12]. These results match those of a previous study by Bello et al., which reported sensitivity, specificity, PPV, NPV, and diagnostic accuracy rates of 89%, 94%, 89%, 94%, and 92%, respectively [11].

Women above 40 years old who present with breast lumps typically undergo a mammogram - a standard procedure - to rule out cancer [13]. Out of 323 cases studied involving both mammography and biopsy, the correlation between the BIRADS score and HPE diagnosis provided noteworthy details. Combining BIRADS-2 and 3, the study revealed that 85.7% of these cases confirmed an absence of malignancy (Table 1). This aligns with a study by Goyal et al., wherein 91.2% of cases proved benign [13]. Significantly, only 23.5% of the cases categorized under BIRADS-4 demonstrated malignancy based on HPE. This falls between Goyal et al.’s [13] finding of a higher 76.9% malignancy and a lower 14.41% identified by Ezeana et al. [14]. In the cases classified under BIRADS-5, 89.5% proved malignant upon HPE, slightly less than Goyal et al.’s study, which reported a 100% malignancy rate in a similar classification [13]. However, a strong positive correlation with a p-value <0.001 was found between the BIRADS scores and HPE diagnoses, a finding analogous to Goyal et al.’s observations [13].

Despite inconsistencies in the literature regarding MRI’s specificity, it has been reported to effectively identify BC in both asymptomatic and symptomatic high-risk patients [13,15]. In our study, MRI demonstrated superior sensitivity, specificity, PPV, and accuracy rates. However, mammography showed the highest NPV. Moreover, there was a significant positive correlation between MRI BIRADS scores and HPE, with a p-value of less than 0.001. Comparing the various radiological modalities used, as shown in Table 1, MRI proved to be superior. While mammography demonstrated higher sensitivity and NPV, US exhibited better specificity, PPV, and accuracy rate. However, these differences were minor for the most part.

Corroborating our study, both Aristokli et al. and Ravert et al. reported MRI to have the highest sensitivity in comparison to mammography and US [15,16]. It is a well-established fact that MRI BC screening is highly effective, particularly for high-risk women, due to its sensitivity in detecting small tumors [5]. Contrarily, both Aristokli et al. and Ravert et al. stated that mammography had the highest specificity at 85.5% [15,16], which opposed our findings. Our study found the highest PPV in MRI at 92.8%, while the best NPV was displayed by mammography. Echoing our study, Ghaemian et al. observed mammography to have a higher sensitivity than US (72.7%, 68.9%), whereas US had a higher specificity (48.6%, 43.9%) [17]. A meta-analysis by Wang et al. explored whether US could be an alternative screening method for BC [18]. Despite this, they found, similar to our results, that mammography still held a higher pooled sensitivity (81% compared to 65%). Hence, they concluded that US is not a sufficient alternative.

The significance of BIRADS-4 scoring can vary greatly. BIRADS-4 lesions exhibit suspicious characteristics but are not sufficient to conclude a malignant interpretation. Malignancy rates range widely, with 4A having the lowest likelihood and 4C being highly suggestive of malignancy [19]. This study focused on all BIRADS-4 cases, regardless of their sub-classification, as these data were missing for a large portion. In US, a significant majority of BIRADS-4 cases (84.2%) were benign. This aligns with Liu et al.’s findings, where 62.7% of BIRADS-4 cases were benign. Similarly, Yoon et al. reported only 18.6% of BIRADS-4 cases as malignant [20]. The present study found an even higher percentage of benign cases in mammography (76.5%), aligning with the findings of Ezeana et al., who reported 85.59% benign results in CNB [14]. Like US and mammography, MRI also showed a significant number of benign BIRADS-4 cases (86.6%). No English language studies discussing the correlation between BIRADS-4 and MRI were found.

In addition to diagnosis, determining the tumor size through radiologic information is a pivotal step for therapeutic planning [4]. This study attests to the accuracy of US, mammography, and MRI in estimating the size of breast tumors by evaluating their compliance with the pathological dimensions of the surgical specimens (Figure 3A-3C). Our findings indicate MRI has the highest conformity with the size of the excised tumor, while mammography comes last. Moreover, we identified the greatest size underestimation with US and the maximum overestimation with mammography. Likewise, Azhdeh et al.’s analysis of the size measurements of 84 breast lesions demonstrated that MRI had the best rate of agreement, with a percentage of 82.1%, whereas mammography scored low at 64.3%. However, they found, unlike our study, an overestimation with MRI at 80%. Yet, they concurred with our findings that US had the highest underestimation rate at 80% [21].

Receptor status and molecular subtyping are crucial in predicting tumor behavior. We hypothesized that more aggressive molecular subtypes might display more suspicious imaging characteristics. To test this theory, we correlated the three principal molecular subtypes (luminal, HER-2 enriched, and basal-like) with the BIRADS classification. Notably, our results showed a significant correlation only in MRI scans where the basal subtype had a higher percentage of BIRADS-5 (malignant) cases. The association was statistically significant, with a p-value of 0.04.

Moreover, identifying a correlation between radiological features like calcification, internal vascularity, spiculation, and molecular subtypes might enable early, noninvasive identification of each subtype. However, due to the limited sample size, MRI was excluded from this study. The association did not achieve statistical significance in US. Contrarily, in a study conducted by Huang et al., a significant connection was established between the luminal subtype and microcalcifications (p = 0.002), spiculation (p = 0.001), and high vascularity (p = 0.004) [22]. Likewise, Algazzar et al. found a notably strong relationship between ultrasonic microcalcifications and the HER-2 enriched subtype (38.5%), as opposed to HER-2 negative (8.5%) (p = 0.02) [23]. Similar to our study, Algazzar and colleagues found no significant differences concerning mass margins. Conversely, the spiculation of margins observed through mammography was significantly associated with the luminal type (p = 0.023), compared to the HER-2 enriched and basal subtypes, at 12.2% and 21.9%, respectively.

Limitations

This study has certain limitations. First, it was conducted at a single center. Second, the possibility of bias due to the influence of previous imaging reports on radiologists cannot be ruled out due to the study’s retrospective nature. Moreover, the limited sample size for MRI cases reduces the significance of the corresponding interpretation. To reinforce these results, a larger-scale, prospective, multi-center study is required.

## Conclusions

MRI proved to be the most precise imaging technique, exhibiting the highest rates of sensitivity, PPV, and accuracy. It was also unrivaled in estimating the size and was the sole modality linked with the basal subtype. Meanwhile, mammography was notably associated with spiculated margins in the luminal molecular subtype of BC compared to the other modalities.
